# Unusual sites of hydatid disease: report of two cases of dumbbell formations

**DOI:** 10.11604/pamj.2020.36.109.24034

**Published:** 2020-06-19

**Authors:** Younes Dkhissi, Badreeddine Alami, Meryem Haloua, Moulay Youssef Alaoui Lamrani, Meryem Boubbou, Mustapha Mâaroufi

**Affiliations:** 1 Radiology Department of Hassan II University Hospital, Fez, Morocco,; 2 Faculty of Medicine and Pharmacy of Fez, Sidi Mohammed Ben Abdellah University, Fez, Morocco

**Keywords:** Vertebral hydatidosis, ecchinococcosis, parasitosis, spine, hydatid cyst

## Abstract

Hydatidosis is a zoonosis caused by *Echinococcus granulosus*. Humans are accidentally contaminated by ingesting the parasite´s eggs mainly released through the faeces from infected dogs. Hydatidosis affects the bone in 0.5 to 2% of cases, with 44% of these cases involving in the spine. Vertebral hydatidosis is rare and it represents the most frequent and most dangerous form of bone involvement. This manifestation is extremely delicate, difficult to correctly identify and manage. The authors report two cases of vertebral hydatidosis revealed by medullar compression and increasing lumbar-radicular pain and functional impotence of lower limbs. Imaging showed multicystic bony lesions in lumbar spine. The extension into the spinal canal and to the perivertebral soft tissue were involved in both cases. We present those two cases to highlight the role of radiological exploration for diagnosis especially with magnetic resonance imaging (MRI) and the importance of monitoring this dangerous pathology.

## Introduction

Hydatid disease is a parasitic infection caused by the larval or adult form of the *Echinococcus granulosus* tapeworm [1]. This is a cosmopolitan anthropozoonosis very common in rural areas. Hydatidos commonly affects the liver and/or the lung. Bone involvement is rare even in endemic countries, hydatic disease of bone is accounting for 0.5 to 2% of all localizations. Vertebral hydatidosis is characterized by a long clinical latency: the infestation can occur in childhood and may be discovered many years later. This condition is more common in young adults, the average age varies between 10 and 30 years with male predominance. The isolated vertebral involvement remains asymptomatic, the radicular pain is the expression of neurological compression [2]. The difficulty remains in the long silent progressive evolution, in the extension of lesions and the frequency of recurrences. We report two cases of invasive vertebral hydatidosis to illustrate the contribution of imaging in the diagnosis and to discuss the therapeutic and prognostic attributed to it.

## Patient and observation

**Figure 1:**
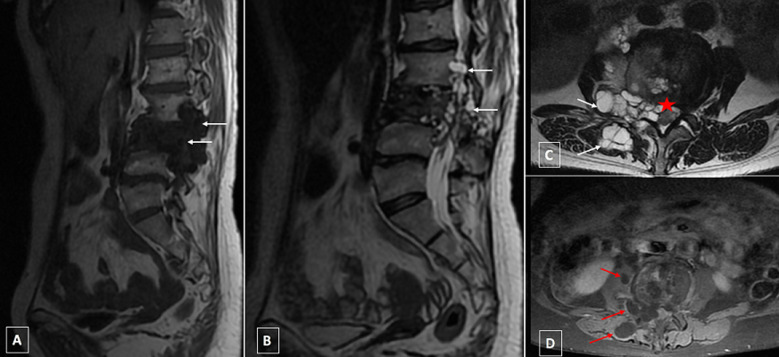
lumbar MRI in sagittal (A,B) and axial sections (C,D) respectively: A (T1WI), B and C (T2WI), D (post gadoliniuim injection) showing multiple cysts (white arrows) at L3 level presenting low signal on SE T1 WI (A) and high signal on SE T2 WI (B) non enhanced after gadolinium injection (D), with extension into the spinal canal and compression of the cauda equina (star) and into the peri vertebral soft tissue (red arrow): dumbbell formation according to Braithwaite and Lees classification

**First case presentation:** a 28-year-old female, of rural origin, complained of weakness and numbness of the lower limb for 25 days. There was no history of trauma, fever, vomiting, altered sensorium or loss of consciousness. The interrogation revealed the presence of sphincter disorders. On examination, the patient was afebrile, conscious and alert. There was no cranial nerve deficit. Neurological examination revealed osteo-tendinous reflexes abolished with flacid tone and bilateral sensory deficit. Biological examinations revealed neither inflammatory syndrome nor eosinophilia. MRI of the spine showed multiple cysts at L3 level with extension into the spinal canal at L2 and L4 levels compressing the cauda equina and to the peri-vertebral soft tissue, presenting low signal on SE T1 weighted images (WI) and high signal on SE T2 WI without any enhancement after gadolinium injection (Figure 1). The other additional examinations did not reveal other localizations of the disease either at liver or lung. The patient underwent medical treatment.

**Figure 2:**
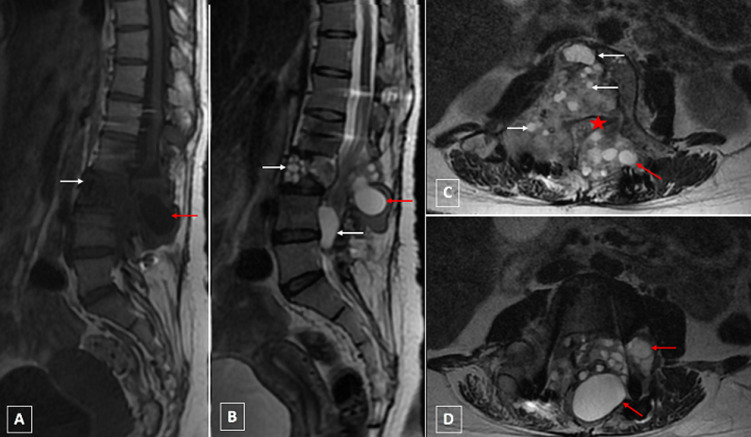
lumbar MRI in sagittal (A,B) and axial sections (C,D) respectively: A (T1WI) and B,C,D (T2WI) showing at L3 level expansible heterogenous lesion with multiple cysts presenting low signal on SE T1 WI (A) and high signal on SE T2 WI (B,C,D), with extension into the spinal canal and compression of the cauda equina (star) and into the peri vertebral soft tissue (red arrow): dumbbell formation according to Braithwaite and Lees classification

**Second case presentation:** a 34-year-old female of rural origin, with occasional contact of dogs, had three years history of lumbar-radicular pain caused by spine hydatid cystic lesions at L3 level and was treated with medical treatment and surgery: corpectomy on L3, disc decompression and stabilization with screws. The parasitological examination of the lesions has documented the presence of *Echinococcus granulosus*. Three years later, she complained increasing lumbar-radicular pain and functional impotence of lower limbs. On examination, the patient was afebrile, conscious and alert. There was no cranial nerve deficit. The patient had difficulty standing, the Lasegue´s sign was positive on the right and Mingazzini test is positive on either side, especially on the right and she has right foot dorsal flexion deficit. She was hospitalized and a lumbosacral MRI was performed. The exam had documented expansible heterogenous mass with multiple cysts at L3 level with extension into the spinal canal at L4 and L5 level compressing the cauda equina and to the soft tissue. The lesions presented low signal on SE T1 WI and high signal on SE T2 WI (Figure 2) without any enhancement after gadolinium injection. The other additional examinations did not reveal other localizations of the disease. It was a recurrence of the same infection, not completely eradicated after the surgery that the patient underwent and medical therapy, followed only for a few months due to lack of adherence to therapy. The patient underwent medical treatment.

## Discussion

The bone involvement of hydatid disease is rare accounting for 0.5-2% of cases [2]. The spine is the most frequent and severe localization, it represents 44% of bone damage. The vertebral involvement is secondary to hematogenous dissemination. The frequency and distribution of the spinal levels involvement in decreasing order are as follows: dorsal localization is the most frequent (80%), followed by lumbar and sacral localization (18%), cervical localization is much rarer [3]. Clinically, symptoms occur only at a late stage of injury, except for the primitive intra-spinal forms. Once present, they manifest by pain, soft tissue swelling or neurological symptomatology associated to a late-occuring spinal deformity. The biological tests reveal hypereosinophilia, but it is inconsistent since it is present in only 25% of cases and it is not specific. Immunoelectrophoresis proves to be the technique of choice due to its specificity by revealing the precipitation of the antigen arc-5 characteristic of hydatidosis [4]. Hydatic serology contributes to post-operative monitoring [4]. Standard X-ray may objectify spinal deformities: kyphosis, scoliosis or gibbosity, with evidence of multilocular osteolysis without periosteal reaction and without bone condensation. The appearance is that of a bone erosion in «honeycomb». Bone condensation would be present only in case of additional osteitis [5]. The ultrasound helps to analyze the soft tissues and shows a collection with central hyperechoic areas. It can also be used to search for other associated visceral localizations. The computed tomography (CT) better analyzes bone damage by showing low density center-bone images that are not enhanced after contrast injection. It can easily and clearly detect bone cortical rupture, erasure of the pedicle and enlargement of the neural foramen and blurring of the costo-transverse joint [5].

**Figure 3:**
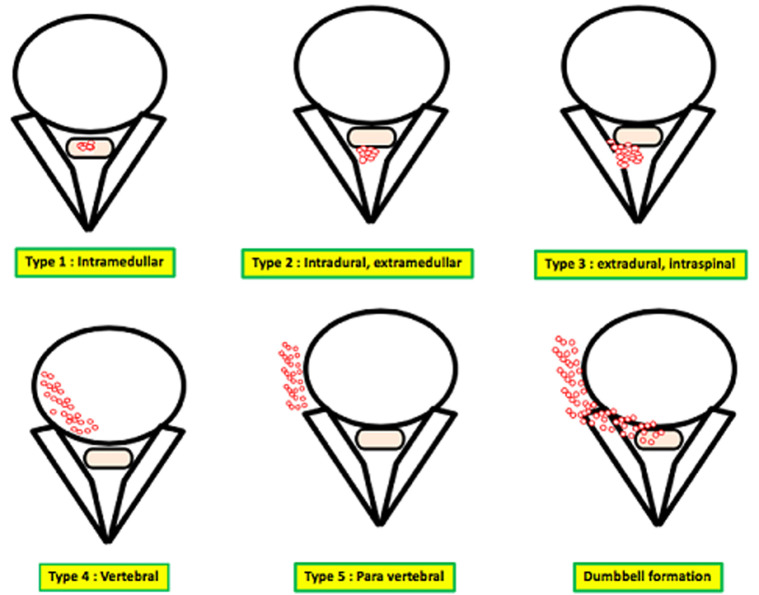
classification of spinal cystic echinococcosis according to the Dew/Braithwaite and Lees classification (type 1-5) and ‘dumbbell’ formation, modified

The CT helps also to evaluate the soft tissues involvement, the presence of the collections. The MRI is currently considered the technique of choice, it is non-invasive and provides a better contrast resolution and multiplanar slices [6]. The MRI shows an oval mass, most often circumscribed without septa, of variable size, with a low signal intensity on T1 weighted images (WI) and a high signal intensity on T2 WI. This lesion is non-enhanced after injection of gadolinium and does not show perilesional edema. MRI also shows soft tissue involvement, best analyzed on coronal and sagittal slices. Braithwaite and Lees [7] proposed a classification into 5 types of vertebrate hydatidosis (Figure 3): type 1: intramedullar cyst; type 2: intradural and extramedullar cyst; type 3: intraspinal and extradural cyst; type 4: vertebral cyst; type 5: paravertebral cyst. They also described the dumbbell formation, which occurs when the vesicles inside the spinal canal extend outside of the neuroforamen, which corresponds to both cases reported. The diagnosis of certainty is made by anatomopathological examination of a surgical excision piece or after percutaneous biopsy. The radioguided biopsy would expose the patient to a risk of spread along the route. Successful treatment of spinal hydatid disease requires careful neuroradiological evaluation, aggressive surgical intervention and this plus adjuvant chemotherapy in some cases. The initial treatment of choice is surgical excision for neural decompression and excision of the lesion depending on the location and the extent of the lesion [8]. Strict follow-up is critical in the management of these patients and regular MRI scans should be done during the postoperative period in order to ensure that any recurrence is detected early [9].

## Conclusion

The vertebral hydatidosis is dangerous by its insidious evolution and its late discovery. It is considered a very aggressive lesion because of the extension of the lesions and its constant recurrence. The literature recurrence ranges from 30% to 100% [10]. The diagnosis is difficult and based on the confrontation of clinical, biological, radiological and histological data. The presence of other visceral localizations strengthens the diagnosis. It must be monitored regularly by imaging especially the CT or even better the MRI.
